# Editorial: Inter-cellular Electrical Signals in Plant Adaptation and Communication

**DOI:** 10.3389/fpls.2018.00643

**Published:** 2018-05-15

**Authors:** Simon Gilroy, Kazimierz Trebacz, Vicenta Salvador-Recatalà

**Affiliations:** ^1^Botany, University of Wisconsin-Madison, Madison, WI, United States; ^2^Department of Biophysics, Maria Curie-Sklodowska University, Lublin, Poland; ^3^Ronin Institute for Independent Scholarship, Montclair, NJ, United States

**Keywords:** ion channel, plant electrophysiology, plant stress, inter-cellular signaling, phloem

Molecular studies suggest that the transition into multi-cellularity took place ~1 billion years ago, after the fungi, animal, and plant lineages had separated (Sanderson, 2003; Peterson and Butterfield, 2005). Although the selective pressures to evolve multi-cellularity are far from understood, comparative studies of two volvocine algae, the colonial *Volvox* and the unicellular *Chlamydomonas* suggest that it was a gradual, multi-step process that involved genetic innovations. Among the first multi-cellularity genes were those that encode extracellular matrix proteins that bind cells together, since *Volvox* has them, but *Chlamydomonas* does not (Prochnik et al., 2010). The increase in cell number and cell types would not have been possible without additional genetic innovations that made it possible for the thousands, millions, and even billions of cells that compose these multicellular organisms to coordinate their activities and cooperate to produce responses to environmental stimuli.

One of the most successful mechanisms to coordinate the activities of distally located cells, tissues, and organs is the electrical impulse, which was first studied in frogs in the 1790s by Galvani and Volta (Galvani, 1791; Pera, 1992). Similar, yet slower electrical impulses were also described in plants a century later by (Burdon Sanderson, 1873), although their relevance was not recognized immediately, being regarded as an anecdotal feature limited to a few exotic species. It is now accepted that most, if not all, plants routinely use electrical and chemical signals that quickly bring into communication distally located cells, yet most aspects of these signals' underlying mechanisms still remain mysterious. With the purpose of promoting this under-researched area of biology and fostering a dialogue between the different perspectives from which it is studied, we invited a diversity of scientists who work on molecular, cellular, and systemic aspects of inter-cellular signals, to contribute their work to this Research Topic. The result is this volume, which contains a diversity of review and original research articles that bring new ideas and elements for reflection, and expand the state-of-the-art of inter-cellular electrical and chemical phenomena, from the ion channel (Ghosh et al.), to the cellular scale (Huang et al.; Niu et al.), and systemic perspectives (Stolarz and Dziubinska; Paulmann et al.).

The review by Szechynska-Hebda et al. set forth an interesting hypothesis to explain the propagation of light-induced systemic electrical signals that involves cellular structures in chloroplasts named “stromules.” Stromules are membrane extensions that connect different chloroplasts, and are proposed to operate as electrical platforms that allow the propagation of these light-related signals. Also in this review, the authors discuss a crosstalk between electrical signaling, as well as calcium and ROS waves, phytohormones, gene expression, and pressure changes in the xylem. In addition, they discuss the genetic basis of the electrophysiological responses to plant illumination, focusing on genes that encode ion channels and membrane transporters that are behind these responses.

A hypothesis and theory article by Brauchi and collaborators also emphasize the genetic bases plant electrophysiological phenomena, in this case of the inter-cellular signals between different cell types, specifically between vascular and non-vascular cells (Canales et al.). Interestingly, *in-silico* analyses of tissue-specific expression of various ion channels have led them to suggest that long- and short-distance systemic electrical signals use different cellular routes. Further, they propose a working model for understanding the cellular pathways and mechanisms by which electrical signals travel from the stimulated peripheral areas to the input region of the phloem, and from the phloem to peripheral cells in effector tissues, one that requires the participation of both the apoplast and plasmodesmata.

Niu et al. demonstrate the involvement of nitric oxide (NO), Ca^2+^ and calmodulin (CaM) in adventitious root formation, which is an important aspect of plant strategy to alleviate osmotic stress. They propose a complex molecular mechanism that involves sequenced activation of NO, Ca^2+^/CaM signaling pathways leading to protection of the photosynthetic apparatus and stimulation of the antioxidant defense system. In another study that also focuses on Ca^2+^ signaling pathways, Huang et al. ask how subcellular compartmentation of the Ca^2+^ signal may play a role in imposing specificity on signaling response systems. By targeting a heterologously expressed Ca^2+^ binding protein to buffer Ca^2+^ changes in specific subcellular locales, they show that nuclear and cytosolic Ca^2+^ changes are independently regulated. However, when assaying downstream events such as alterations in gene expression, changes in both compartments are shown to be required for the complex coordinated responses to osmotic and salt stresses.

Here, Stolarz and Dziubinska bring new light into the classical problem of the sunflower's circumnutation movement, a phenomenon driven by ion and water fluxes, with a study that clarifies the connections between action and spontaneous potentials and glutamate-induced potentials. A different study by Paulmann et al. shows an indirect yet relevant contribution of the phloem's role as an electrical signaling network to determine the susceptibility of barley to viral infection. By altering the phloem's anatomy, the virus blocks the electrical message that would alert of its presence.

Two research articles show new findings that underscore an unsuspected degree of sophistication of the plant electrical responses to environmental stimuli. On one hand, in their report of a new, protective role of local electrophysiological responses over the photosynthetic process under high temperature stress, Sukhov et al. show significant electrophysiological responses of pea leaves to increases of only 1°C above the background temperature. On the other hand, Ghosh et al. show that plants can consistently distinguish between touch and sound vibration. Both research articles support the notion that plants are much more sensitive to external stimuli than previously thought, and respond to these with strong and finely tuned responses. In the first case (Sukhov et al.), in the form of variation potentials that propagate between cells; in the second case (Ghosh et al.), by increasing or decreasing the expression of similar yet distinct mechanosensitive ion channels.

All together, the articles included in this Research Topic bring new theory and data to this exciting research area that hopefully will spark future studies that help us to understand how these rapid communication systems aid plants in overcoming their lack of mobility in the complex, dynamic, and often hostile environments in which they thrive.

## Author contributions

All authors listed have made a substantial, direct and intellectual contribution to the work, and approved it for publication.

## Conflict of interest statement

The authors declare that the research was conducted in the absence of any commercial or financial relationships that could be construed as a potential conflict of interest.
